# Physiological Chemistry

**Published:** 1902-09

**Authors:** John A. Miller

**Affiliations:** Buffalo, N. Y.


### PROGRESS IN MEDICAL SCIENCE.

### Physiological Chemistry.

By JOHN A. MILLER, Ph. D., Buffalo, N. Y.

### METT'S METHOD OF ESTIMATING PEPTIC ACTIVITY.

Alexander A. Samojloff (*Pflüger's Arch.*, 85, *J. Chem. Soc.*, 80.) Mett's method consists in measuring the decrease in length of cylinders of egg white coagulated in capillary tubes, which occurs under the influence of artificial digestive juices. The method is recommended as an accurate one. The law that peptic activity is proportional to the square root of the amount of pepsin present is shown to be true, except when the amount of pepsin is very great; with high concentrations, the figures found are less than those calculated.

### VARIATION OF THE QUANTITY OF THIOCYANATE CONTAINED IN HUMAN SALIVA AND ITS CAUSES IN HEALTH AND DISEASE.

Jul. A. Grober (*Chem. Centr.*, 1901; *J. Chem. Soc.*, 80.) Potassium thiocyanate is found in the human body only in the saliva. (This statement is not true, as it exists in both the blood and the urine.—J. A. M.) It is not formed by the decomposition of the saliva, but is actually secreted, and the quantity diminishes with the duration of the secretion. The quantity present in the saliva is not affected by change of diet in the case of healthy persons or by the use of tobacco by non-smokers. The secretion of the thiocyanate is probably dependent on the condition of the organism in respect to the albumin decomposed and utilised, and where this is small in amount, as in severe cachectic cases, little or no thiocyanate is secreted.

### THE FUNCTIONS OF BILE AS A SOLVENT.

Benjamin Moore and William H. Parker (*Proc. Roy. Soc.*, 1901; *J. Ch. Soc.*, 80.) Bile acts as a solvent in two ways: (1) it dissolves lecithin, and to a less extent cholesterol; it thus aids the excretion of these otherwise insoluble substances by the liver cells, and their carriage to the intestine; (2) it dissolves both free fatty acids and soaps, and thus renders the absorption of these substances easier. The solvent properties are chiefly due to the bile salts, but in the case of the fatty acids and soaps the amount dissolved is greatly increased by the simultaneous presence of lecithin.

FEBRILE CHANGES IN THE CHEMICAL COMPOSITION OF BLOOD.

KARL RITTER VON STEJSKAL (*Chem. Centr.*, 1901; *J. Ch. Soc.*, 80.) The blood of fever patients has been found to contain less albumin, fat, cholesterol, iron, and chlorine, and to yield a smaller quantity of dry substances than normal blood. On the other hand, the quantities of water, calcium, potassium, and ash were in excess of the usual amount, whilst the amounts of lecithin and sodium were normal. The blood serum showed a decrease of albumin, substances soluble in ether, chlorides, and dry substances, but contained a greater quantity of potassium, and yielded a larger ash. The fact that in the febrile state the red corpuscles contain less albumin, lecithin, and cholesterol, but are richer in water and salts can only be due to imbibition of solutions containing salts (plasma) and especially chlorides. The red corpuscles had increased in weight, whilst the plasma had correspondingly decreased.

---

ANALYSIS OF LIQUID OBTAINED FROM A HYDATID CYST OF THE LIVER.

F. MALMÉJAC (*J. Phar.*, 1901; *J. Ch. Soc.*, 80.) The liquid (1012 c.c.) was perfectly colorless, limpid, and of acid reaction, and contained 13 grams of solid matter per litre, of which sodium chloride forms 5.8 grams, urea 2 grams, and calcium oxide 1 gram. In addition small quantities of serum-albumin, sulphates, phosphates, and acetone are present.

---

VARIATIONS IN THE COMPOSITIONS OF THE BILE.

R. L. CRACIUNU (*Compt. Rend.*, 1901; *J. Ch. Soc.*, 80.) In young animals, the solids are more abundant than in old ones. In thin animals, the solids are more abundant than in fat ones. The amounts of fat and lecithin in the bile increase with age.

---

VARIATIONS IN THE AMOUNT OF THIOCYANATE IN HUMAN SALIVA.

E. C. SCHNEIDER (*Am. J. Physiol.*, 1901; *J. Ch. Soc.*, 80.) In smokers, the average percentage of potassium thiocyanate is 0.013; in non-smokers, 0.003. The amount diminishes on prolonged stimulation of the salivary glands. The parotid saliva is always richer than the submaxillary saliva of the same person at the same time.

### PROTEIDS OF THE THYMUS.

W. Huiskamp (*Zeit. Physiol. Chem.*, 1901; *J. Ch. Soc.*, 80.) Nucleo-histon is the most abundant proteid in thymus; it comprises 69.4 per cent. of the total proteids; 18.7 per cent. of nucleo-proteid, and 11.9 per cent. of other proteids are present. The characters and composition[1] of nucleo-histon and nucleo-proteid are fully described, together with their compounds with calcium, magnesium and sodium. Nucleo-histon contains 3.7 and nucleo-proteid, 0.9 per cent. of phosphorus. The influence of these proteids on the coagulation of blood plasma was found to be much the same as described by Hammarsten with solutions of fibrinogen.

### ARTIFICIAL MODIFICATIONS OF TOXIN.

James Ritchie (*J. Hyg.*, 1901; *J. Ch. Soc.*, 80.) Tetanus toxin under the influence of hydrochloric acid readily loses its virulent properties, but the capacity of producing immunity remains. The less poisonous substances produced are probably toxoids. Sodium hydroxide or carbonate has similar power. Ricin is resistant to the action of hydrochloric acid, but when toxicity is destroyed, the capacity of producing immunity remains. Abrin is also resistant to the acid, but is relatively susceptible to sodium hydroxide; much the same is true of diphtheria toxin, although the power of producing immunity remains as in the other cases.

### HIPPURIC ACID METABOLISM IN MAN.

Carl Lewin (*Chem. Centr.*, 1901; *J. Ch. Soc.*, 80.) A healthy man secretes daily from 0.1 to 0.3 gram of hippuric acid. This amount is increased by feeding on dextrose, by increase in intestinal putrefaction, or by administration of foods rich in nuclein, such as sweetbread, this being, however, attributed to the increase of putrefaction in the intestines, since nucleic acid does not produce the effect. In gout and diabetes, the amount is normal; in febrile conditions, in kidney disease, and in perityphlitis the amount of hippuric acid rises.

### DIGESTIVE POWER OF GASTRIC JUICE.

Albert Fronin (*Compt. Rend.*, 1901; *J. Ch. Soc.*, 80.) Variations in the digestive power of gastric juice depends chiefly on the amount of hydrochloric acid. Proteid food increases the secretion of pepsin.

---

NON-PERMEABILITY OF THE WALL OF THE URINARY BLADDER.

OTTO COHNHEIM (*Zeit. Biol.*, 1901; *J. Ch. Soc.*, 80.) In the intestinal wall, the stream of liquid is in one direction only; in the peritoneum, diffusion resembles that which occurs through parchment paper. In the urinary bladder, the liquid contained within it alters no more than it would in a glass vessel. The bladder wall is not a diffusion membrane, or a semi-permeable membrane, and is not permeable to water. These differences furnish evidence of the "physiological component" in the movement of liquid through living animal membranes. As soon as these three membranes are poisoned with sodium fluoride the differences between them disappear, and they all behave like diffusion membranes.

